# Cyanobacteria-Derived Proline Increases Stress Tolerance in *Arabidopsis thaliana* Root Hairs by Suppressing Programmed Cell Death

**DOI:** 10.3389/fpls.2020.490075

**Published:** 2020-12-14

**Authors:** Alysha Chua, Orla L. Sherwood, Laurence Fitzhenry, Carl K.-Y. Ng, Paul F. McCabe, Cara T. Daly

**Affiliations:** ^1^Department of Science, Waterford Institute of Technology, Waterford, Ireland; ^2^Pharmaceutical and Molecular Biotechnology Research Centre (PMBRC), Waterford Institute of Technology, Waterford, Ireland; ^3^Eco-Innovation Research Centre (EIRC), Waterford Institute of Technology, Waterford, Ireland; ^4^UCD School of Biology and Environmental Science, University College Dublin, Dublin, Ireland; ^5^UCD Centre for Plant Science, University College Dublin, Dublin, Ireland; ^6^UCD Earth Institute, University College Dublin, Dublin, Ireland

**Keywords:** programmed cell death (PCD), proline, biofertiliser, cyanobacteria exometabolites, root hair assay (RHA), *Nostoc muscorum*, plant stress tolerance

## Abstract

Nitrogen-fixing heterocystous cyanobacteria are used as biofertilizer inoculants for stimulating plant growth but can also alleviate plant stress by exometabolite secretion. However, only a small number of studies have focused on elucidating the identity of said bioactives because of the wide array of exuded compounds. Here, we used the root hair assay (RHA) as a rapid programmed cell death (PCD) screening tool for characterizing the bioactivity of cyanobacteria *Nostoc muscorum* conditioned medium (CM) on *Arabidopsis thaliana* root hair stress tolerance. We found that heat-stressed *A. thaliana* pre-treated with *N. muscorum* CM fractions exhibited significantly lower root hair PCD levels compared to untreated seedlings. Treatment with CM increased stress tolerance by suppressing PCD in root hairs but not necrosis, indicating the bioactive compound was specifically modulating the PCD pathway and not a general stress response. Based on documented *N. muscorum* exometabolites, we identified the stress-responsive proline as a compound of interest and strong evidence from the ninhydrin assay and HPLC indicate that proline is present in *N. muscorum* CM. To establish whether proline was capable of suppressing PCD, we conducted proline supplementation experiments. Our results showed that exogenous proline had a similar effect on root hairs as *N. muscorum* CM treatment, with comparable PCD suppression levels and insignificant necrosis changes. To verify proline as one of the biologically active compounds in *N. muscorum* CM, we used three mutant *A*. *thaliana* lines with proline transporter mutations (*lht1*, *aap1* and *atprot1-1::atprot2-3::atprot3-2*). Compared with the wild-type seedlings, PCD-suppression in *lht1*and *aap1* mutants was significantly reduced when supplied with low proline (1–5 μM) levels. Similarly, pre-treatment with *N. muscorum* CM resulted in elevated PCD levels in all three mutant lines compared to wild-type seedlings. Our results show that plant uptake of cyanobacteria-derived proline alters their root hair PCD sensitivity threshold. This offers evidence of a novel biofertilizer mechanism for reducing stress-induced PCD levels, independent of the existing mechanisms documented in the literature.

## Introduction

Cyanobacteria are adaptable organisms found in aquatic and terrestrial ecosystems. Their ability to inhabit most environments is attributed to the diverse range of exuded metabolites, termed exometabolites that can have antiviral, antibacterial, antifungal, antitumoral and anti-inflammatory properties (Singh et al., 2005; Jaiswal et al., 2008; Prasanna et al., 2010). In agriculture, nitrogen-fixing heterocystous cyanobacteria are often used as biofertiliser inoculants to stimulate plant growth. For example, *Anabaena*-inoculated wheat seedlings had improved shoot length, grain weight and phytohormone (cytokinin and indole-3-acetic acid) levels compared to untreated controls (Hussain and Hasnain, 2011). Biofertiliser inoculants contain little macro- and micro-nutrients as they are catalysts for mobilizing nutrients into metabolically accessible forms that are otherwise unavailable to plants (Kennedy, 2008). Depending on the functional characteristics of the inoculant, biofertilizers can either directly or indirectly provide yield gain. Direct benefits make essential macronutrients available for plant growth via nitrogen fixation and phosphate solubilization, while indirect benefits rely on assorted mechanisms to safeguard against abiotic and biotic stresses (Barreto et al., 2011).

*Nostoc* is a genus of blue-green, N_2_-fixing bacteria which can be free-living but can form symbiotic relationships with fungi (Rai et al., 2000) and several plant species, such as the hornwort *Anthoceros punctatus* (Campbell and Meeks, 1989) and the angiosperm *Gunnera* (Rasmussen et al., 1994). *Nostoc muscorum* is the model organism for studying heterocyst differentiation but it cannot differentiate into akinetes and hormogonia, unlike other taxonomically defined *Nostoc* species (Meeks et al., 2002). A wide range of compounds have been found in *N. muscorum* extracellular filtrate, termed conditioned media (CM), such as amino acids (Picossi et al., 2005; Pernil et al., 2008), exopolysaccharides (EPS) (Mehta and Vaidya, 1978), auxin (Mirsa and Kaushik, 1989; Karthikeyan et al., 2009), abscisic acid (ABA) (Maršálek et al., 1992) and phenolics and alkaloids (Abdel-Hafez et al., 2015). Previous work (unpublished data) showed that *Nostoc muscorum* sp.7120 (hereafter, *N. muscorum*) CM suppresses root hair programmed cell death (PCD) in heat-stressed *Arabidopsis* seedlings, but the identity of these pro-survival signals were not identified. Considered the model organism for studying heterocyst differentiation, *N. muscorum* has undergone many name changes over the years. *Nostoc* sp. strain PCC 7120 was originally named *Nostoc muscorum*, before being classified as *Anabaena* and finally renamed as *Nostoc* sp. strain PCC 7120 based on DNA–DNA hybridization data and short tandem repeated repetitive fingerprinting (Svenning et al., 2005).

Chen and Dickman (2005) have shown that exogenous proline inhibits stress-induced PCD levels in *Colletotrichum trifolii* and *Saccharomyces cerevisiae* by quenching reactive oxygen species (ROS). Proline might have a similar role in plants as it is a stress-responsive amino acid that indirectly scavenges ROS by stimulating the plant antioxidant defense and glyoxalase system (Hossain et al., 2014, 2016; Rejeb et al., 2014). Studies have reported the presence of proline in *N. muscorum* extracellular medium (Picossi et al., 2005; Pernil et al., 2008) and plants have three root-localized transporters for importing proline: amino acid permease 1 (AAP1), lysine-histidine transporter 1 (LHT1) and proline transporter (ProT) (Lehmann et al., 2010).

AAP1 is an intermediate-affinity transport system for neutral amino acids (i.e., proline), glutamate and aspartate (Svennerstam et al., 2011). Expressed in the *Arabidopsis* root epidermis and tips, AAP1 imports extracellular amino acids into the vascular system for long-distance transport (Lee et al., 2007). LHT1 is a broad-specificity, high-affinity transporter for histidine, acidic and neutral amino acids like proline (Hirner et al., 2006). During the early developmental stage, LHT1 is expressed in the rhizodermis of emerging and lateral roots to import soil amino acids (Hirner et al., 2006). In later stages, LHT1 supplies leaf mesophylls with xylem-derived amino acids and is expressed throughout the root epidermis and tips, leaf mesophyll, stem, petals and sepals (Hirner et al., 2006). In contrast to both general amino acid transporters, the ProT subfamily only imports proline, but can also transport stress-induced compounds such as glycine betaine and *γ-*aminobutyric acid (Schwacke et al., 1999; Grallath et al., 2005). Three subfamily members have been characterized in *Arabidopsis* (AtProT1, AtProT2 and AtProT3) and they are expressed differently all over the plant (Lehmann et al., 2011). Phloem-localized AtProT1 is expressed in the vascular tissue of leaves, petioles, roots, flowers, siliques, and stems (Rentsch et al., 1996). However, AtProT1 expression is absent in root tips and has weak expression levels in emerging lateral roots. Conversely, AtProT2 expression is mostly present in the root cortex and epidermis, while AtProT3 expression is only found in leaf epidermis (Grallath et al., 2005). As external proline can be assimilated by *Arabidopsis*, it is a possible candidate for the bioactive PCD-suppressing effect noted in *N. muscorum* CM.

PCD is activated by developmental and environmental factors as it plays an important role in vegetative and reproductive tissue development (Kacprzyk et al., 2011; Daneva et al., 2016). However, plant cells also undergo PCD to mitigate stress effects, such as hypoxia (Lenochová et al., 2009), salinity (Shabala, 2009), drought (Nguyen et al., 2009), UV overexposure (Ferreyra et al., 2016), heavy metal exposure (Xu et al., 2013), heat (Vacca et al., 2004) and pathogen infection (Lam et al., 2001). PCD is a methodical process of cellular destruction characterized by the distinctive retraction of the cytoplasm; this active and interruptible process is driven by cellular Ca^2+^ influx (Kacprzyk et al., 2017). Conversely, necrosis is associated with uncontrolled cell death that occurs when cells cannot withstand overwhelming cellular stress (Reape et al., 2008). As substantial differences in signaling, morphology and regulation exist between PCD and necrosis (Kacprzyk et al., 2017), assessing the stress response only using a single parameter loses context as to whether cells are dying by activated PCD or uncontrolled necrotic death. Therefore, it is important to differentiate between both death modes to paint an accurate picture of plant cell death studies across different research groups (Reape et al., 2008; Reape and McCabe, 2013).

In this study, we used the root hair assay (RHA) to demonstrate the PCD-suppressing bioactivity of *N. muscorum* CM. 5-day old *Arabidopsis* seedlings were pre-treated with *N. muscorum* CM fractions and heat stress applied. Using a combination of viability staining and death morphologies, the RHA was used to quantify the root hair stress response in terms of cell viability, PCD, and necrosis. This was done to test if the bioactive compound was affecting the PCD pathway or modulating a general stress response. Based on documented *N. muscorum* exometabolites, the stress-responsive amino acid proline was highlighted as a compound of interest. Proline was detected in *N. muscorum* CM using the ninhydrin assay and HPLC. Following that, we confirmed the bioactivity of proline by assessing how exogenous proline affected heat-shocked wild-type *Arabidopsis* seedlings. Finally, we also compared the performance of *Arabidopsis* proline transporter mutants (*lht1*, *aap1* and *atprot1-1::atprot2-3::atprot3-2*) against stress-induced PCD levels of wild-type seedlings pre-treated with proline before heat shock exposure. To the best of our knowledge, this study is the first instance to show that proline enhances *in vivo* root hair stress tolerance by modifying the PCD activation threshold.

## Materials and Methods

### Growth and Sterilization Procedures for Seedlings

Seeds of *Arabidopsis thaliana* L. ecotype Columbia (Col-0) were soaked in 20% bleach (Domestos^®^ disinfectant: sodium hypochlorite −4.5 g per 100 g) aseptically, the bleach solution was removed, and the seeds were rinsed five times with sterilized deionised water (SDW). Sterilized seeds (15–20) were placed in a straight line on germination medium comprising ½-strength Murashige and Skoog (MS) agar plates, adjusted to pH 5.8 with NaOH and solidified with 6 g/L Duchefa^®^ plant agar. The composition of the ½ MS plates were as follows: 0.0125 mg/L CoCl_2_.6H_2_O, 0.0125 mg/L μM CuSO_4_.5 H_2_O, 18.4 mg/L FeNaEDTA, 3.10 mg/L H_3_BO_3_, 0.415 mg/L KI, 8.45 mg/L MnSO_4_. H_2_O, 0.125 mg/L Na_2_MoO_4_.2 H_2_O, 4.30 mg/L ZnSO_4_.7 H_2_O, 0.166 g/L CaCl_2_, 0.085 g/L KH_2_PO_4_, 0.950 g/L KNO_3_, 0.090 g/L MgSO_4_, and 0.825 g/L NH_4_NO_3_. Plates were stratified at 4°C for 24 h to synchronize germination and placed vertically under light (33 μmol m^–2^ s^–1^, 16-h light: 8-h darkness) in a 21°C growth chamber to germinate seeds. *Arabidopsis* seedlings developed sufficient root hair density after five days of growth and were then used for stress assays.

### Heat Stressing of *Arabidopsis thaliana* Seedlings

*Arabidopsis* seedlings (5-day old) were transferred using sterile forceps into individual wells of a sterile 24-well plate (Sarstedt^®^ Tissue Culture Plate) containing 1 ml SDW. Seedlings were handled with care during the transfer process to avoid mechanical damage to root hairs and elevated background death levels. 24-well plates were sealed using autoclave tape, placed in a Grant SUB Aqua Pro 26 water bath already stabilized at the desired heat stress temperature (25, 35, 45, 50, 55, 65, 75, or 85°C), and heat stressed for 10 min. Seedlings were returned to the 21°C growth chamber and scored 14–16 h after stress application to allow PCD morphology to fully develop. The RHA was used to quantify the stress response in terms of viability, PCD and necrosis as described by Hogg et al. (2011).

### Assessing the Plant Stress Response Using PCD Morphology and Viability Stain

**FIGURE 1:**
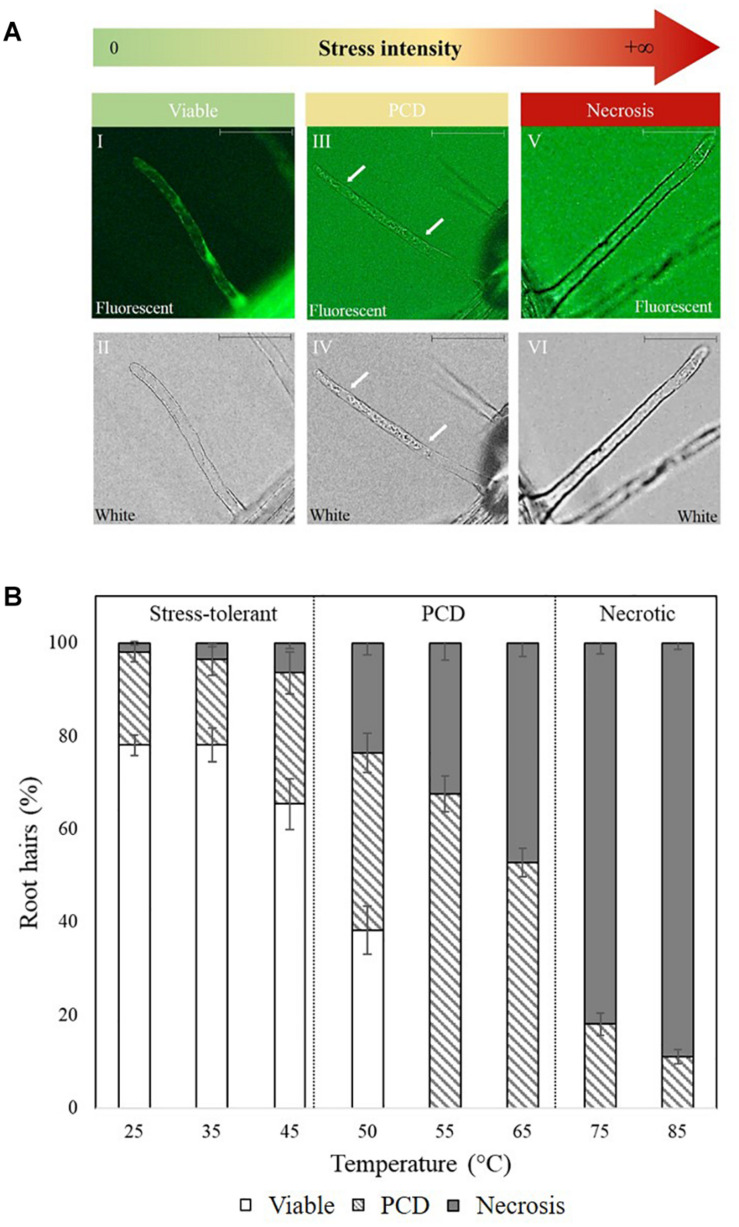
**(A)** Cell morphology of FDA-stained root hairs of heat shocked *Arabidopsis* seedlings under fluorescent (I, III, and V) and white light (II, IV, and VI). Viable root hairs are FDA positive and exhibit fluorescence (I and II – no heat shock), PCD root hairs are FDA negative and have a retracted cytoplasm indicated by white arrows (III and IV – 50°C heat shock), and necrotic root hairs are FDA negative but do not have a retracted cytoplasm (V and VI – 80°C heat shock). Scale bars: I–VI 10 μm. **(B)** Effect of heat stress on *Arabidopsis* root hair viability and cell death (PCD and necrosis) levels. The bars represent viable (white), PCD (hatched) and necrosis (gray) root hairs, each expressed as a percentage of cell mode over total number of root hairs. Values are means ± SE (*n* ≥ 12) and represent the merged results of 3 experiments.

Direct scoring of root hairs relied on a combination of the fluorescein diacetate (FDA) viability stain and cell corpse morphology (PCD and necrotic root hairs) as visual indicators. *Arabidopsis* seedlings were placed on microscope slides, stained with a 0.001% w/v FDA solution for 2 min and examined using an Olympus BX61 microscope with a FITC filter. The root hairs were scored as follows: (A) viable when exhibiting positive FDA staining, (B) PCD if exhibiting a negative FDA stain and retracted cytoplasm and (C) necrotic if the FDA stain is negative and the protoplast is not retracted (Figure 1A; Hogg et al., 2011). At least 100 root hairs were scored per seedling to give an accurate representation of the levels of viable cells and dead cells (PCD + necrosis). The proportions of cells in each state is expressed as percentage of the total number of analyzed root hairs.

For additional evidence (Supplementary Figure 1) to confirm heat-exposed root hairs were dead and not merely dehydrated, whole trichoblast cells were stained with Evans Blue indicating the whole cell was dead, and not just the cytoplasmic extension that forms the root hair. To this end, a 5 day old *A. thaliana* Col-0 seedling was heat stressed at 49 °C for 10 min, incubated for 30 h under constant illumination and then stained for 1 h in 0.25% Evans Blue stain.

### Profiling Bioactive *Nostoc* spp. Exometabolites in Conditioned Medium (CM)

*Nostoc muscorum* cultures from the Pasteur Culture Collection of Cyanobacteria Paris, France (PCC 7120 available for order from the following link: https://catalogue-crbip.pasteur.fr/recherche_catalogue.xhtml), were grown in BG11 media at 25 °C (light intensity of 30 μmol m^–2^ s^–1^, 16-h light: 8-h darkness) and shaken at 110 rpm under sterile conditions to maintain axenic cultures. Culture growth was monitored by measuring optical density (Myers et al., 2013), chl-*a*, and carotenoid concentration by methanol extraction according to Zavřel et al. (2015). *N. muscorum* CM was harvested in the deceleration phase [OD_730_ (1.17), chl-*a* (14.14 μg ml^–1^), carotenoid (3 μg ml^–1^)] after two cycles of centrifugation (Eppendorf Centrifuge 5810 R^®^) at 3000 × *g* for 20 min. After each cycle, the supernatant was collected, and the pellet discarded to eliminate leftover cells. The resulting cell-free supernatant was sterile-filtered through a 0.45 μm PES filter; half of the filtered supernatant was autoclaved (121°C for 15 min) while the other half remained unautoclaved. *Nostoc* CM fractions (autoclaved and non-autoclaved) were diluted in BG11 at various concentrations and screened for PCD-suppressing bioactivity by pre-treating *Arabidopsis* seedlings with CM fractions for 3 h in 24-well plates, followed by 50°C exposure for 10 min in the water bath. Viability, PCD, and necrosis levels were scored 14–16 h later using the RHA.

### Ninhydrin Assay

A modified ninhydrin-based protocol (Bates et al., 1973) that did not rely on the use of toluene was adapted from Claussen (2005), who used it to determine stress-induced proline levels in tomato plants. The protocol from Claussen (2005) was used to quantify proline levels in autoclaved and non-autoclaved CM. A mixture containing 400 μl of CM, 400 μl of glacial acetic acid, and 400 μl ninhydrin mixture (2.5% ninhydrin dissolved in 6:3:1 ratios of glacial acetic acid, SDW and 85% orthophosphoric acid) were vortexed and heated at 100°C for 1 h in a block heater (Stuart^®^ SBH130D). The reaction was terminated by incubation at 21°C for 5 min, followed by quantification at 546 nm using an Ultrospec 2000^®^ spectrophotometer. Proline concentration was determined from a proline standard curve.

### Detection of Amino Acids Using Reverse-Phase HPLC

A modified protocol from Heinrikson and Meredith (1984) and Kwanyuen and Burton (2010) was adapted to detect amino acids in *N. muscorum* CM. Phenyl isothiocyanate (PITC) was chosen as the precolumn derivatization agent as it reacts with both primary and secondary amines such as proline and hydroxyproline, unlike other derivatizing agents such as *o*-phthalaldehyde (Walker and Mills, 1995). Amino acid standards (TCI Chemicals), each corresponding to 1.5 mM, were prepared individually and in a mixture in 0.1 M HCl. *N. muscorum* CM was harvested in the deceleration phase [OD_730_ (1.67), chl-*a* (33.19 μg ml^–1^) and carotenoid (9.77 μg ml^–1^) after two cycles of centrifugation at 3000 × *g* for 20 min. After each cycle, the supernatant was collected and sterile-filtered through a 0.45 μm PES filter. Following that, 40 μl of the amino acid mixture or 200 μl of filtered *N. muscorum* CM sample was added to 100 μl of coupling buffer (acetonitrile: pyridine: triethylamine: H_2_O, 10:5:2:3) and dried under vacuum by rotary evaporation (ScanSpeed 32^®^) at 85°C. Derivatization was performed by adding 20 μl of a 7:1:1:1 ratio mixture of ethanol: water: triethylamine: PITC (v/v). The resultant mixture was incubated for 20 min in the dark at room temperature to form phenylthiocarbamyl derivatives (PTC-amino acid) that were quantified using reverse-phase HPLC. Samples were then dried under vacuum at 35°C because of PTC amino acid sensitivity to light and high temperature. The pellet was re-suspended in 100 μl of 4 mM sodium phosphate (pH 7.4) and 2% (v/v) acetonitrile and was injected into a Symmetry^®^ C18 column (3.99 mm × 15 cm, 5 μm particle size) in a HPLC system (Agilent Technologies 1200 Series). An injection volume of 14 μl was used for the amino acid mixture, while 70 μl was injected for the *N. muscorum* CM sample. The mobile phase consisted of two solvents: Solvent A was 70 mM sodium phosphate (pH 6.55, adjusted by NaOH) and 2% acetonitrile (v/v); solvent B comprised 50% (v/v) acetonitrile. The following step-wise gradient was used to separate the amino acid peaks: 0–1 min [0% Solvent B; 5.5–7 min (15% B); 8.5–13.5 min (30% B); 14 min (35% B); 15.5 min (42% B); 16 min (43% B); 20 min (60%); 22 min 0% B]. Absorbance of the PTC-amino acid adducts was monitored at 254 nm.

### Evaluating the Effect of Exogenous Proline and *N. muscorum* CM in Root Hairs of Wild-Type and Mutant *Arabidopsis* Lines

Two proline solutions were established in BG11 at identical concentrations previously measured in autoclaved CM (1.94 μM) and non-autoclaved CM (1.83 μM), with the former solution autoclaved at 121°C for 15 min. Both proline solutions (autoclaved and non-autoclaved) were diluted in BG11 at various concentrations. Five-day old *Arabidopsis* seedlings were incubated for 3 hr in the proline solutions and seedlings were then heat stressed at 50°C for 10 min. We chose 50°C as it is a stress intensity that can either give rise to high levels of PCD or cell survival, depending on the elicitor treatment. Therefore, stress exposure at this viability/PCD inflection point informs us on the effect of various treatments on the perturbation of cell death or survival signaling pathways. Seedlings were returned to the 21°C growth chamber and scored for viability, and death via necrosis or PCD 14–16 h after heat stress application. This protocol was repeated with *Arabidopsis* lines with proline transporter mutations: *lht1* (Hirner et al., 2006), *aap1* (Lee et al., 2007) and the *atprot* triple knockout (*atprot1-1::atprot2-3::atprot3-2)* (Lehmann et al., 2011). Five-day old *Arabidopsis* mutant seedlings were treated with exogenous proline (1, 2, 5, or 100 μM) or fresh 100% *N. muscorum* CM (OD_730_ = 1.43), chl-*a* = 18.9 μg ml^–1^ and carotenoid = 4.67 μg ml^–1^) for 3 h, heat stressed at 50°C for 10 min and returned to the 21°C growth chamber. The RHA was used to score viable, PCD and necrotic root hairs of the mutants after 14–16 h of stress application.

### Statistical Analysis

IBM^®^ SPSS^®^ Version 24 (RRID:SCR_002865) was used to analyze results for significant changes (*p* < 0.05) across elicitor treatment and mutant *Arabidopsis* lines. Statistical tests used include one-way (Tukey or Dunnett *Post-hoc* Test) and two-way ANOVA analysis.

## Results

### The Arabidopsis Heat Stress Baseline Response

Baseline heat stress responses were established in *Arabidopsis thaliana* and two distinctive stress-response thresholds were detected: stress-tolerant, PCD and necrosis (Figure 1B). Most of the root hairs remained viable (65–75%) at 25–45°C, but at 50–65°C, cell death accumulated at greater rates, with PCD being the predominant cell death form. This changed under overwhelming heat stress (75–85°C) as root hairs primarily died by necrosis, instead of PCD. Based on the dose-dependent response, 50°C was identified as the inflection point as it was located at the stress-tolerant/PCD threshold, i.e., the transition border between the majority of root hairs remaining alive versus PCD activation.

### Screening *N. muscorum* CM for PCD-Suppression

The secretion of pro-survival signals into extracellular filtrate have been observed in animal (Barres et al., 1992) and plant cells (McCabe et al., 1997), and similar observations by C.T. Daly (unpublished data) suggest that *N. muscorum* CM also contains pro-survival signals. To identify the pro-survival signals, the RHA was used as a high-throughput system for screening *N. muscorum* CM for PCD-suppressing activity. *N. muscorum* was cultured in a closed batch system and the harvested CM diluted with fresh BG11 to generate a concentration range (20–100%) to determine the optimum CM% for the strongest PCD-suppressing effect at the 50°C inflection point. We used a two-way ANOVA test to analyze if increasing the *N. muscorum* CM concentration and autoclaving fractions would influence stress-induced PCD levels. Each individual variable had a strong significant (*p* < 0.01) effect on PCD levels and the adjusted r-squared value (0.333) shows that 33.3% of the variance in PCD levels can be attributed to autoclave treatment and CM% fraction. The interaction effect was non-significant, *F* (4, 121) = 0.49, *p* = 0.743.

**FIGURE 2:**
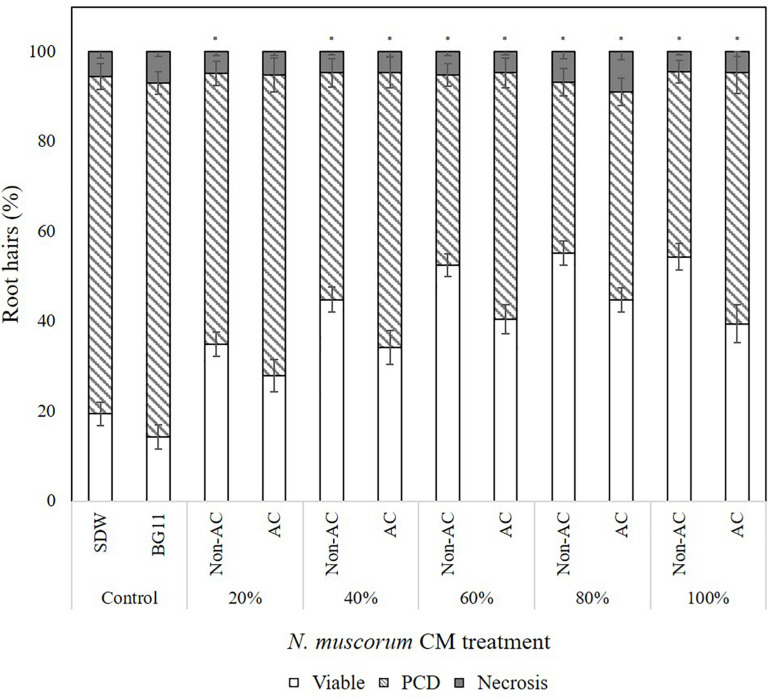
Effect of autoclaved (AC) and non-autoclaved *N. muscorum* CM on *Arabidopsis* root hair viability and death (PCD and necrosis) levels at 50°C heat stress. The bars represent viable (white), PCD (hatched) and necrosis (gray) root hairs, each expressed as a percentage of cell mode over total number of root hairs. Values are the average of *n* ≥ 8 (±SE) and represent the merged results of 3 experiments. (^∗^) marks PCD levels significantly (*p* < 0.05) different from the BG11 control using a Dunnett t-test (Supplementary Table 1).

For example, CM treatment exerted a statistically significant dose-dependent effect, where *F* (4, 121) = 0.49, *p* = 0.000. As shown in Figure 2, the strongest protection effect was offered by 60–100% non-autoclaved CM treatment (38–42% PCD), which equates to an approximate 30% decrease in PCD levels compared to SDW and BG11 (75–78% PCD) controls. All tested *N. muscorum* CM fractions, apart from the 20% autoclaved treatment, were significantly different (*p* < 0.05) from the BG11 control (Supplementary Table 1). Similarly, autoclaved CM treatments resulted in higher mean PCD levels (*M* = 57.1%, *SE* = 1.46) over non-autoclaved CM supplementation (*M* = 46.5%, *SE* = 1.52), where *F* (1, 121) = 25.4, *p* = 0.000. We tested autoclaved and non-autoclaved CM fractions to determine if the PCD-suppressing compound was thermolabile; both treatments suppressed PCD in treated root hairs showing that the major compound was thermostable. However, the PCD-suppression effect in autoclaved *N. muscorum* CM was consistently lower than non-autoclaved CM across all five tested CM concentrations (20–100%). Autoclaved *N. muscorum* CM treated seedlings had significantly (*p* < 0.05) higher PCD levels, with an average difference of 10.6%, compared to their non-autoclaved treated counterparts. Lastly, *N. muscorum* CM treatment shifted the PCD activation threshold, as a proportion of root hairs normally dying by PCD in treated seedlings were now viable. There were negligible changes in necrosis levels across all tested CM concentrations as improvements in viability levels corresponded to increasing PCD suppression.

### Identification and Quantification of Proline as the Compound of Interest

Our findings from the initial *N. muscorum* CM screening process showed that the bioactive compound was thermostable and directly modulating the PCD pathway. This information was cross-referenced with data from a literature review on *N. muscorum* exometabolites and used as input for building a framework to identify possible bioactive candidates (see “Discussion”). The resultant framework highlighted proline as a candidate of interest as it accumulates in plants under abiotic and biotic stress (Abrahám et al., 2010; Hayat et al., 2012). Proline was detected in *N. muscorum* CM using two separate assays: the ninhydrin assay and reverse-phase HPLC. Using the ninhydrin assay, similar proline concentrations (1.83–1.94 μM) were detected in autoclaved and non-autoclaved CM used in the screening experiments, which had insignificant variability once the standard error was considered (Table 1). The ninhydrin assay is a colorimetric method for quantifying proline as ninhydrin produces a distinctive red chromophore when reacting with proline under acidic conditions. While the other proteinogenic amino acids give no color (Friedman, 2004), the ninhydrin assay can overestimate proline levels when high levels of structurally related amino acids are present, as ornithine, D-proline and δ^1^-pyrroline-5-carboxylic acid (P5C) produces a similar red color (Forlani and Funck, 2020).

**TABLE 1:** Quantification of proline in autoclaved and non-autoclaved *N. muscorum* CM [OD_730_ (1.17), chl-*a* (14.14 μg ml^–1^) and carotenoid (3 μg ml^–1^)].

*N. muscorum* Conditioned Media (CM)	Proline concentration (μM)
Autoclaved	1.83 ± 0.26
Non-autoclaved	1.94 ± 0.22

*Values are the average of *n* = 16 (±SE) and represent the merged results of 2 experiments.*

**FIGURE 3:**
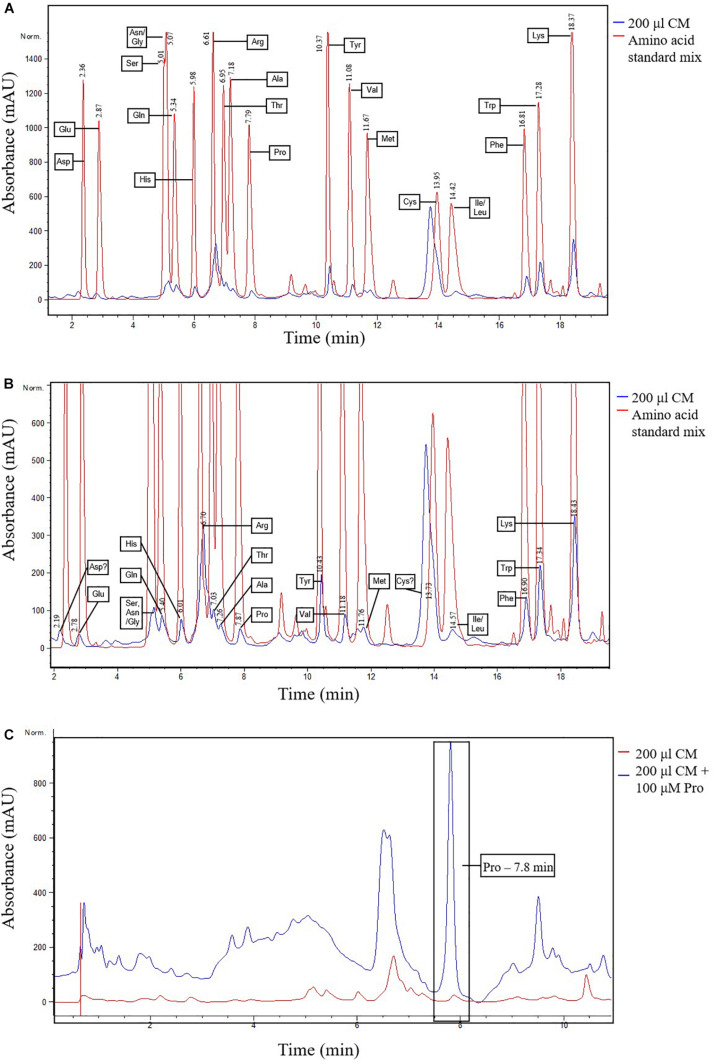
Detection of proline and other amino acids in *N. muscorum* CM sample. **(A)** Overlaid chromatogram of *N. muscorum* CM and amino acid standard mix and **(B)** its close-up view of the individual amino acid peaks. **(C)** Confirmation of the elution of proline at the 7.8 min point by spiking *N. muscorum* CM with an internal 100 μM proline standard. Asp, Aspartic acid, Glu, Glutamic acid, Ser, Serine, Asn, Asparagine, Gly, Glycine, Gln, Glutamine, His, Histidine, Arg, Arginine, Thr, Threonine, Ala, Alanine, Pro, Proline, Tyr, Tyrosine, Val, Valine, Met, Methionine, Cys, Cysteine, Ile, Isoleucine, Leu, Leucine, Phe, Phenylalanine, Trp, Tryptophan, and Lys, Lysine.

For these reasons, a reverse-phase HPLC method was developed for additional evidence of proline and the other amino acids in *N. muscorum* CM. We utilized a different *N. muscorum* CM batch for HPLC analysis, separate from the batch originally used in the bioactivity screening and ninhydrin assay. Older cultures in the deceleration phase were specifically chosen for better amino acid detection sensitivity as culture age significantly influences the composition of cyanobacteria exometabolites (Volk, 2007). Separation of the amino acid standard mixture was achieved under the run conditions, with proline achieving a satisfactory peak resolution of 4.61 with alanine, its adjacent peak (Figure 3A, red lines). 200 μL of *N. muscorum* CM was concentrated (a 5-fold concentration factor) and analyzed for amino acid content. Proline was successfully detected in the 200 μL *N. muscorum* CM sample as a peak (70.73 mAU) that eluted at the 7.87-minute mark (Figure 3B) and its presence demonstrated by spiking the 200 μL *N. muscorum* CM with an internal 100 μM proline standard (Figure 3C). After overlaying the *N. muscorum* sample with the amino acid standard mixture, we detected the following amino acids in *N. muscorum* CM: glutamic acid, serine, asparagine/glycine, glutamine, histidine, arginine, threonine, alanine, tyrosine, valine, methionine, isoleucine/leucine, phenylalanine, tryptophan and lysine.

### Confirming the Bioactive PCD-suppressing Effect of Proline

#### Evaluating the Effect of Exogenous Proline and *N. muscorum* CM in Wild-type (Col-0) *Arabidopsis* Lines

We prepared two proline solutions in BG11, at concentrations identical to that measured using the ninhydrin assay in autoclaved CM (1.94 μM) and non-autoclaved CM (1.83 μM). The former solution was autoclaved at 121°C for 15 min to determine if proline was the thermostable bioactive compound in *N. muscorum* CM. Both proline solutions were diluted across a similar concentration gradient (20–100%) to assess if proline elicits a dose-dependent response. The SDW control was omitted for this series of experiments as past results and statistical analysis (Supplementary Table 1) show that BG11 and SDW treatment results in similar PCD levels, with no bioactive effect noted in treated wild-type *Arabidopsis* seedlings.

**FIGURE 4:**
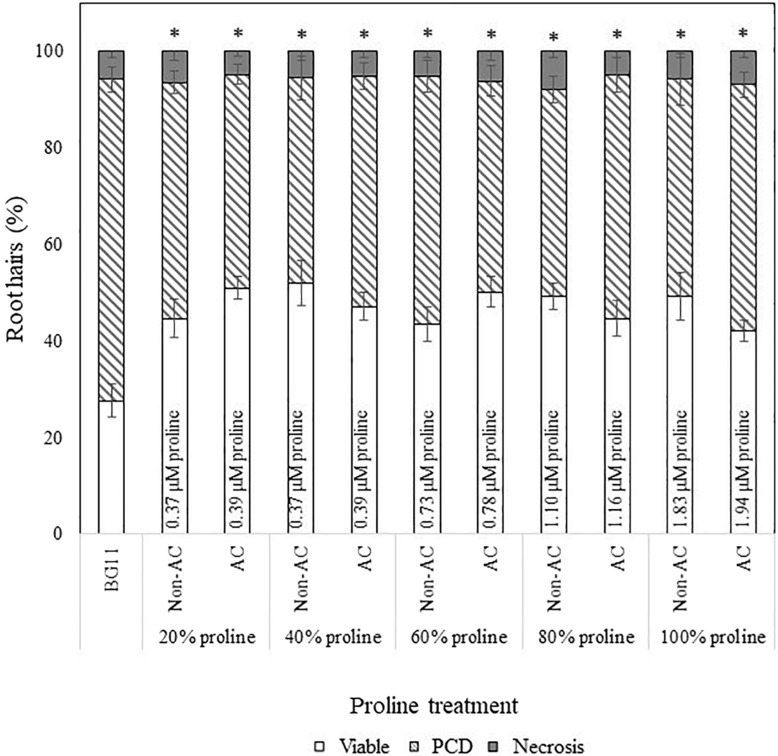
Effect of autoclaved (AC) and non-autoclaved exogenous proline on root hair viability and cell death modes of *Arabidopsis* at 50°C. A proline gradient (20–100%) was made up to assess its similarity to the *N. muscorum* gradient in Figure 2. Solutions labeled ‘100% proline’ corresponded to proline levels previously measured in undiluted *N. muscorum* CM. The remaining gradient (20–80%) was established in BG11, where 80% proline = 80% proline + 20% BG11, 60% proline = 60% proline + 40% BG11, etc., (^∗^) marks PCD levels significantly (*p* < 0.05) different from the BG11 control (Supplementary Table 2). The bars represent viable (white), PCD (hatched) and necrosis (gray) root hairs, each expressed as a percentage of cell mode over total number of root hairs. Values are the average of *n* ≥ 12 (±SE) and represent the merged results of 3 experiments.

We also applied a two-way ANOVA test to assess if proline was thermostable and if exogenous proline treatment exerted a dose-dependent effect. This was done by assessing if autoclave treatment and diluted proline fractions (20–100%) affected stress-induced PCD levels. Both variables did not have a significant effect and the interaction effect was also non-significant, where *F* (4, 115) = 2.14, *p* = 0.08. Autoclave treatment had an *F* ratio of *F* (4, 115) = 0.347, *p* = 0.557, indicating that proline was thermostable. There was no significant (*p* > 0.05) differences between autoclaved (*M* = 46.1%, *SE* = 1.49) and non-autoclaved (*M* = 47.4%, *SE* = 1.57) proline treatments, with an average mean difference of 1.28%. Our results confirmed that proline was thermostable as all tested proline fractions (autoclaved and non-autoclaved) significantly reduced (*p* < 0.05) stress-induced PCD levels of treated *Arabidopsis* seedlings, with up to a 24% mean difference from the BG11 control (Supplementary Table 2). Moreover, proline treated seedlings exhibited a similar stress-response profile as *N. muscorum* CM treatment: treated seedlings had lower stress-induced PCD levels, but negligible changes to necrosis levels (Figure 4), demonstrating the PCD-suppressing ability of proline. Unlike *N. muscorum* CM though, exogenous proline did not inhibit PCD in a dose-dependent manner, where *F* ratio of F (1, 115) = 0.195, *p* = 0.941. Overall, we show that proline was thermostable and suppressed *Arabidopsis* stress-induced PCD levels as necrosis changed negligibly across the entire treatment range.

#### Comparing the Effect of *N. muscorum* CM and Exogenous Proline in Heat-Stressed *Arabidopsis* Seedlings

**FIGURE 5:**
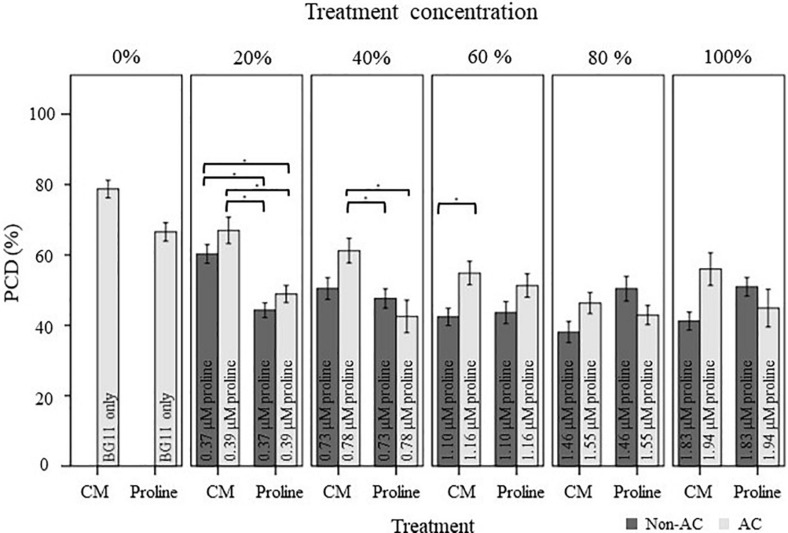
Comparison of the stress-induced PCD levels of autoclaved (AC) and non-autoclaved *N. muscorum* CM and exogenous proline in 50°C-heat shocked *Arabidopsis* seedlings. Samples were diluted in BG11 across a concentration gradient (0–100%). Solutions labeled ‘100% proline’ corresponded to proline levels previously measured in undiluted *N. muscorum* CM, while the remaining solutions were diluted in BG11, where 80% proline = 80% proline + 20% BG11, 60% proline = 60% proline + 40% BG11, etc., Values for each dataset represent the average of *n* ≥ 8 (±SE) and represent the merged results of 3 experiments. Datasets marked with an (^∗^) are statistically different (*p* < 0.05) to each other using a one-way ANOVA Tukey *post-hoc* test (Supplementary Table 3).

We examined the extent of PCD-suppression between *N. muscorum* CM and exogenous proline treatments at each dilution factor from 20–100% (Figure 5). One-way ANOVA analysis at each % of CM/proline fraction revealed two key trends: (1) the greatest variations primarily affected autoclaved *N. muscorum* CM treated seedlings as they had higher PCD levels compared to the other treatments and (2) significant differences (*p* < 0.05) between *N. muscorum* CM and exogenous proline datasets predominantly occurred at the lower concentrations, but largely disappeared at the more concentrated doses (Supplementary Table 3). The largest fluctuation in root hair PCD levels were observed for dilutions of CM and proline solutions in the range of 20–40%, but dilutions from 60–100% did not have significant differences (*p* > 0.05) between *N. muscorum* CM and exogenous proline datasets. This shows that comparable PCD-suppression occurs at concentrated doses between proline-treated root hairs and their corresponding CM fractions – offering preliminary evidence for proline as the bioactive compound.

### Evaluating the Effect of Exogenous Proline and *N. muscorum* CM in Mutant *Arabidopsis* Lines

**FIGURE 6:**
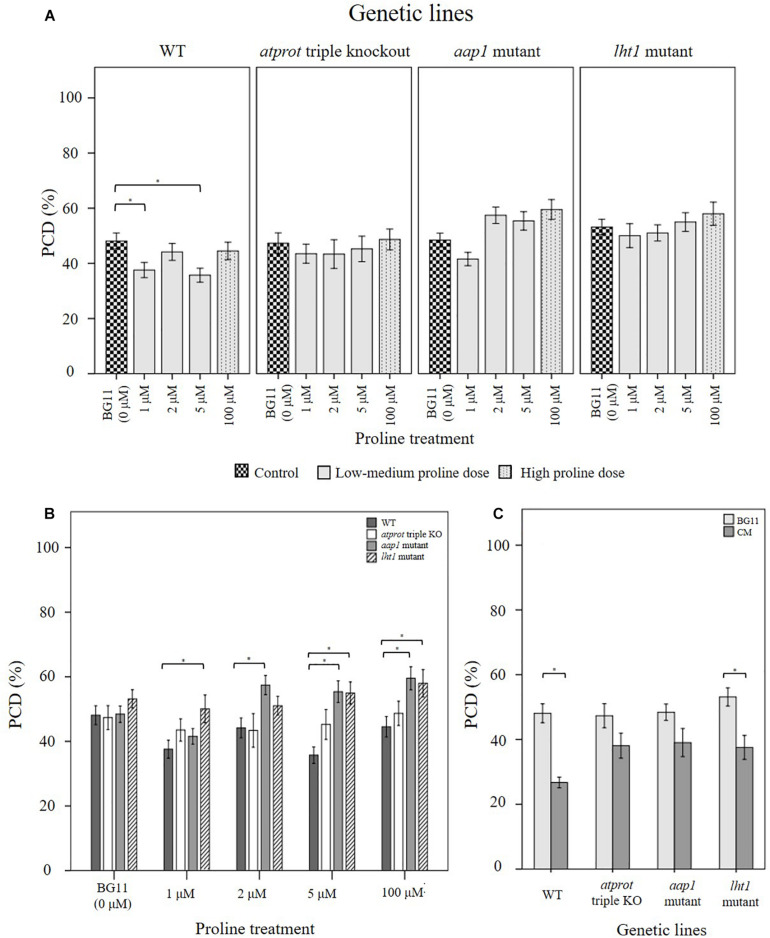
Examining how proline bioactivity differs between 50°C-heat shocked wild-type and proline transporter mutants (*atprot1-1::atprot2-3::atprot3-2*, *aap1*, and *lht1*) upon **(A, B)** proline or **(C)** *N. muscorum* CM treatment. Figures **A and B** are the same data collated into different groupings. Datasets marked with an (^∗^) are statistically different (*p* < 0.05) to each other (Supplementary Tables 4, 5). Values are the average of *n* ≥ 12 (± SE) and represent the merged results of 3 experiments.

We evaluated the stress response profile of three proline transporter mutants against wild-type seedlings after *N. muscorum* CM and exogenous proline treatment. Due to differences in the ages of wild-type seed batches and their storage conditions, we obtained different PCD levels in the BG11 controls in Figures 6A,B (∼50%), compared to a different experimental set in Figure 5 (65–80%). All four *Arabidopsis* lines (wild-type, *lht1*, *aap1* and the *atprot* triple knockout mutant line) were treated with undiluted *N. muscorum* CM (100% CM), low (1 μM), medium (2–5 μM), or high (100 μM) proline levels and two controls (SDW and BG11). For clarity, the SDW dataset is omitted here as it has no significant (*p* > 0.05) differences with the BG11 control but is displayed in Supplementary Table 4.

Wild-type seedlings benefited the most out of the four tested *Arabidopsis* lines, whether they were treated with exogenous proline or *N. muscorum* CM (Figure 6). An overall trend of lower stress-induced PCD levels were observed with proline treatments from 1–5 μM, and significant reductions (*p* < 0.05) were noted with 1 μM and 5 μM compared to the BG11 control (Supplementary Table 4). Biological variability probably accounts for weak effect at 2 μM proline as the −3.94% reduction was not sufficiently high enough to be significant. *N. muscorum* CM-treated wild-type plants also had the lowest PCD levels (26.7%) of all four *Arabidopsis* lines which equated to approximately 21.4% lower than its untreated control. However, a cytotoxic effect was noted when proline was supplemented at high (100 μM) doses; proline lost its protective effects as stress-induced PCD levels rose to 44.5% and was not statistically (*p* = 0.894) different from the BG11 control seedlings. A similar effect took place when proline was supplemented at 1000 μM, resulting in 54% PCD (data not shown), showing that excessive proline doses have a cytotoxic effect.

There were a number of interesting observations from the mutant supplementation study: (1) the PCD-suppressing effects of proline was attenuated in proline transporter mutants, (2) the *atprot* triple knockout mutant displayed a stress phenotype more similar to wild-type seedlings, (3) differences between *atprot* triple knockout mutant with *aap1* and *lht1* mutants only becomes apparent at different proline doses, and (4) priming mutants with *N. muscorum* CM eliminated differences between mutants.

First, proline transporter mutants responded differently to exogenous proline treatment (Figure 6A). Statistical analysis confirmed that all three mutant lines had no significant differences (*p* > 0.05) across the entire 1–100 μM proline treatment compared to their respective BG11 controls (Supplementary Table 4). This was reflected in the stability of their PCD levels, as the biggest mean differences from their respective BG11 controls were insignificant, e.g., the *atprot* triple knockout mutant (4.6%), *lht1* (4.8%), and to a lesser extent *aap1* (11.1%). Thus, the PCD-suppressing effects observed in proline-treated wild-type seedlings were lost in the proline transporter mutants.

Nevertheless, we also observed a marked difference between the stress-response of proline-specific (*atprot* triple knockout) and the general amino acid transporter (*lht1* and *aap1*) mutants. The *atprot* triple knockout mutant displayed a stress phenotype more akin to wild-type seedlings, unlike the *lht1* and *aap1* knockout mutants (Figure 6B). The *atprot* triple knockout mutant had negligible changes to PCD (43–48%) levels across all proline treatments and were not statistically different (*p >* 0.05) from the wild-type seedlings at identical proline doses (Supplementary Table 5). In contrast, *aap1* and *lht1* mutants shared a similar stress phenotype, with higher PCD levels than wild-type and the *atprot* triple knockout mutant.

Next, the variances between the *atprot* triple knockout and both general amino acid transporter mutants were apparent at medium (2–5 μM) and high (100 μM) proline doses, but had little differences at low (1 μM) proline treatment (Figure 6B). At low proline levels, all three mutants had similar PCD levels (41–50%) to each other but a different pattern emerged when they were supplied with medium and high proline doses. The *atprot* triple knockout mutant only had slightly higher PCD levels (up to a 9.5% increase) than wild-type lines at medium and high proline doses. In contrast, both general amino acid transporter mutants were more susceptible to death and displayed up to a 19% increase in PCD levels compared to wild-type seedlings. This was reflected in statistical analysis showing that *aap1* and *lht1* mutants were significantly different (*p* < 0.05) from wild-type seedlings at 5 μM and 100 μM proline doses (Supplementary Table 5).

Finally, priming with *N. muscorum* CM eliminated the phenotypic difference between the *atprot* triple knockout and general amino acid transporter mutants as we detected similar PCD levels (35–39%) across all three mutant lines (Figure 6C). The CM bioactive effect was weaker in mutant lines as CM-treated mutants had a higher average of PCD levels (8–12%) than wild-type seedlings, but only the *app1* mutants were significantly different (*p* = 0.032) (Supplementary Table 5). It appears than even in mutant lines, the accompanying bioactive compounds in *N. muscorum* CM were likely acting synergistically to exert a stronger PCD-suppressing effect than proline alone.

## Discussion

**FIGURE 7:**
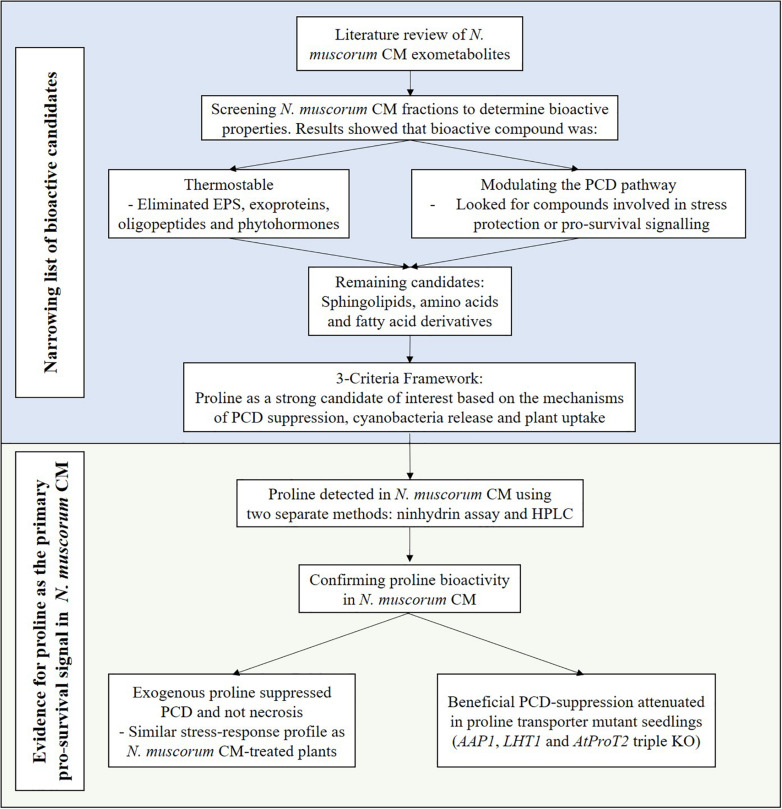
Workflow for identifying the pro-survival signal in *N. muscorum* CM.

Survival signals such as platelet-derived and insulin-like growth factors can inhibit PCD in animal cells (Barres et al., 1992) and similar observations have been noted in plants as McCabe et al. (1997) showed that carrot cell CM inhibits stress-induced PCD at low cell densities. Previous work by C.T. Daly (unpublished data) suggest that *N. muscorum* CM contains pro-survival signals that exert a similar bioactive effect in *Arabidopsis* root hairs but attempts to identify the compound are difficult as *N. muscorum* exudes a broad range of exometabolites. Here, the RHA was used as a rapid screening tool to characterize *N. muscorum* CM bioactivity on root hair stress tolerance in terms of viability, PCD and necrosis. By using this high-throughput method, we developed a workflow for identifying and assessing the validity of the main bioactive compound in *N. muscorum* CM, as summarized in Figure 7.

To achieve this, we first established the baseline stress response in untreated wild-type (Col-0) *Arabidopsis* root hairs and identified similar thresholds (Figure 1B), first reported by Hogg et al. (2011). For example, root hairs primarily underwent PCD when subjected to heat stress within the temperature range in which cells die predominantly of PCD. At this stage, root hairs either survive if the cellular protective mechanisms can repair the heat-induced damage or activate PCD if the response is insufficient. By identifying the PCD threshold, 50 °C was chosen as the set-point for screening *N. muscorum* CM. Beyond this temperature, root hairs cannot survive and crossing the necrotic threshold causes necrosis to replace PCD as the primary cell death mode because of excessive cellular damage. This biphasic cell death motif concurs with past *in vivo* (Hogg et al., 2011) and *in vitro* (Lennon et al., 1991; McCabe et al., 1997; Mammone et al., 2000; Burbridge et al., 2007) studies showing that the severity of an insult governs the fate of the cell.

In the initial *N. muscorum* CM screening, our results yielded a few key observations. Firstly, the main PCD-suppressing bioactive compound in *N. muscorum* CM was thermostable as autoclaving did not attenuate the pro-survival signal in treated *Arabidopsis* seedlings. Additional thermolabile bioactives were likely acting in synergy with the main bioactive to suppress PCD as stress-induced PCD levels in root hairs were consistently lower in non-autoclaved CM treated seedlings than their autoclaved CM-treated counterparts. Furthermore, *N. muscorum* secretes EPS into their growth medium (Mehta and Vaidya, 1978) and autoclaving sugars with phosphate (BG11 medium contains high K_2_HPO_4_ concentrations) generates cytotoxic products (Finkelstein and Lankford, 1957; Wang and Hsiao, 1995). Therefore, the diminished capacity of autoclaved CM to suppress PCD was likely a combination of the destruction of synergistic thermolabile bioactive compounds and leftover cytotoxic by-products from the autoclaving process. Lastly, the main bioactive compound in *N. muscorum* CM shifted the PCD threshold instead of decreasing necrosis levels. Distinguishing whether the pro-survival signal affects PCD or necrosis is important as modulation of a general stress response affects necrosis, while treatments targeting the PCD pathway itself lowers PCD levels, but not necrosis (Reape et al., 2008). Thus, the main bioactive compound appeared to be directly affecting the PCD pathway and not a general stress response. Collectively, these observations were used to narrow the list of bioactive candidates as a literature review of *N. muscorum* exometabolites showed that they can be grouped into the following categories: exoproteins (Oliveira et al., 2015), EPS (Mehta and Vaidya, 1978), amino acids (Picossi et al., 2005; Pernil et al., 2008), the phytohormones auxin (Mirsa and Kaushik, 1989; Karthikeyan et al., 2009) and ABA (Maršálek et al., 1992), phenolics and alkaloids (Abdel-Hafez et al., 2015), and fatty acid derivatives (Abdel-Hafez et al., 2015).

Compounds were grouped into orders of importance based on their ability to withstand thermal degradation. This eliminated thermolabile groups such as EPS and exoproteins as primary candidates, while phytohormones (Sigma-Aldrich, 2019) and phenolics (Zainol et al., 2009; Igual et al., 2011; Sharma and Gujral, 2011) were considered secondary candidates as thermal processing results in a significant loss of biological activity. After filtering candidates based on their thermostability, the remaining candidate groups were amino acids, and fatty acid derivatives. By considering three factors (mechanism of PCD inhibition, cyanobacteria release and plant uptake), proline arose as a promising candidate as it accumulates in plants under abiotic and biotic stress (Abrahám et al., 2010; Hayat et al., 2012).

Proline protects against oxidative damage by upregulating the antioxidant defense and glyoxalase system (Hossain et al., 2014, 2016; Rejeb et al., 2014). Growth medium supplementation of 5–20 mM proline upregulated flux through the influential H_2_O_2_-detoxifying ascorbate-glutathione (AsA-GSH) cycle and methylglyoxal-detoxification pathways for increased tolerance to salt (Hoque et al., 2008; Hossain and Fujita, 2010) and cold stress (Kumar and Yadav, 2009). Both pathways are linked by GSH, a redox buffer that modulates the stress acclimation response as efficient GSH recycling through the glyoxalase and AsA-GSH cycle lessens the oxidative load on plant cells (Yadav et al., 2005; Hossain et al., 2016). This holds important significance for our work as ROS signaling is complicit in PCD activation in animals and plants (Doyle et al., 2010; Wang et al., 2013; Gutiérrez et al., 2014). Moreover, exogenous proline was effective at quenching ROS and inhibiting PCD in *Colletotrichum trifolii* and *Saccharomyces cerevisiae* under a range of stress elicitors (UV radiation, salt, heat, H_2_O_2_ and paraquat) (Chen and Dickman, 2005). Proline appears to have an important protective role in suppressing ROS-mediated PCD (Chen and Dickman, 2005) and here we report a similar effect *N. muscorum* CM has in plants.

The evidence points towards cyanobacteria-derived proline as the main bioactive ingredient as proline buffers against oxidative damage by indirectly scavenging ROS in stressed plants (Hossain et al., 2014, 2016; Rejeb et al., 2014). Additionally, unspecific leakage of proline through the *natH* transporter enables its release by *N. muscorum* cells into the extracellular medium (Picossi et al., 2005). Finally, plants have three amino acid transporter subfamilies (two general and one proline-specific) that can import proline from their surroundings into plant roots (Lehmann et al., 2010). On the whole, this offers a working paradigm as to how cyanobacteria-derived proline can prime the root hair stress response in heat-shocked *Arabidopsis* seedlings.

Following this, we used two assays to provide evidence for the presence of proline in *N. muscorum.* The ninhydrin assay was first used to quantify the proline levels, but the assay may provide false positives or overestimate the proline concentration in CM as it side-reacts with hydroxyproline and pipecolic acid. Therefore, we used the HPLC for additional proof and found similar retention times of the putative proline peak in the CM sample with both the proline standard and proline-spiked CM samples. Collectively, our results offer strong evidence for proline in the CM. We then followed-up with two sets of experiments to determine if proline was the main bioactive compound in CM. In the first experimental series, we supplied wild-type *Arabidopsis* seedlings with exogenous proline at the concentrations measured in *N. muscorum* CM. Our results showed that exogenous proline elicited a similar stress response profile to *N. muscorum* CM treatment by increasing viability levels by inhibiting PCD, but not necrosis. All ten proline fractions (autoclaved and non-autoclaved) significantly reduced the proportion of root hairs initiating PCD in treated *Arabidopsis* seedlings compared to the BG11 control. Moreover, autoclaving proline did not attenuate its PCD suppressing effects, showing that the main bioactive compound was highly thermostable. Interestingly, exogenous proline did not suppress PCD in a dose-dependent manner as seen in the *N. muscorum* CM fractions, at least not in the current tested range. Proline likely acts in synergy with the accompanying bioactive exometabolites in *N. muscorum* CM to exert a stronger PCD-suppressing effect than individual proline treatments alone. Moreover, we observed a cytotoxic effect when seedlings were supplemented with overtly high proline doses. This was reminiscent of past studies reporting the cytotoxic effects of over-supplying proline in *Distichlis spicata* suspension cultures (2–10 mM) (Rodriguez and Heyser, 1988) and *Arabidopsis* (5–20 mM) (Hare et al., 2002) and rice seedlings (5–10 mM) (Chen and Kao, 1995). For example, Hellmann et al. (2000) showed that *Arabidopsis* (ecotype C24) plants developed lesions after prolonged incubation on agar plates containing 200 mM proline (48 h).

Next, we investigated how *Arabidopsis* wild-type and proline transporter (*lht1*, *aap1* and *atprot* triple knockout) mutants responded towards proline and *N. muscorum* CM treatment. A proline gradient was used as all three transporter subfamilies possess varying affinities for proline; heterologous *Saccharomyces cerevisiae* expression showed that *ProT* transporters had the lowest proline affinity, e.g., AtProT1 (427 μM), AtProT2 (500 μM), AtProT3 (999 μM), AAP1 (60 μM), and LHT1 (10 μM) (Lehmann et al., 2010). By comparing the performance of the mutant lines against wild-type seedlings, we sought to confirm if proline was one of the major bioactive ingredients in *N. muscorum* CM, while discerning the role of each transporter in stress tolerance.

Our results showed that the beneficial PCD-suppressing proline effect seen in wild-type seedlings was lost in all three mutant lines. Similarly, the bioactive effect of CM treatment was weaker across the mutant lines, although only the *aap1* mutant was statistically different (*p* = 0.032) from the wild-type seedlings. Under low to medium proline doses, PCD was inhibited in wild-type seedlings, but this effect was lost in the mutant lines, especially in *aap1* and *lht1* mutants. Statistical analysis confirmed this as all three mutants had no significant (*p* > 0.05) PCD deviations across all proline treatments, compared to their respective BG11 controls. A similar root hair stress-response profile was observed when the mutants were treated with *N. muscorum* CM: all mutants had higher PCD and lower viability levels than wild-type seedlings under identical treatments. As the mutant lines have an impaired ability to import proline, their subsequent higher root hair PCD levels offered further evidence that proline was an important bioactive compound in *N. muscorum* CM.

It must be noted that we did not assess the mutants at other temperatures in the absence of CM or proline treatments. As of now, no study has investigated the possible effect of the amino transporter mutations on temperature sensitivity; current studies include exposure of *aap1* mutants to toxic levels of amino acids (2–100 mM) (Lee et al., 2007), *atprot* mutants to salt stress (Lehmann et al., 2011) and *lht1* mutants grown with elevated inorganic nitrogen levels (Hirner et al., 2006). In future experiments, it would be interesting to explore if the mutations would have an impact on temperature sensitivity, i.e., if the threshold for PCD induction would shift to another temperature because of possible deleterious effects from impaired proline transport. Also, we acknowledge that stressed plants can elevate internal proline levels by upregulating proline biosynthesis instead of relying on external proline uptake. However, prokaryotic studies show that proline uptake is preferred over biosynthesis if the osmolyte is already readily available (Roesser and Müller, 2001). Similar findings have been shown in plants; osmotic stressed maize (Verslues and Sharp, 1999) and salt stressed barley roots (Ueda et al., 2007) contain elevated proline levels, despite low levels of proline biosynthesis in the root tips. This was further underscored by elevated *HvProT* expression in barley root cap cells under salt stress and minimal pyrroline-5-carboxylate synthetase (P5CS1) activity (Ueda et al., 2001). Taken together, this implies that stressed plants also prefer importing proline compared to the biosynthesis route as it enables metabolic resources to be channeled towards other cell protective mechanisms for improved survival rates (Verslues and Sharma, 2010).

Although all three mutants did not respond to the PCD-suppressing effects of proline, a distinct root hair stress-response profile was observed between the general amino acid transporter (*lht1* and *aap1*) and *atprot* triple knockout mutants. The *atprot* triple knockout mutant displayed a stress phenotype more reminiscent to wild-type seedlings than the mutant lines. Lehmann et al. (2011) showed that single, double and *atprot* triple knockout mutants had no discernible changes between the shoot size, root length and flowering time, compared to wild-type seedlings grown under axenic conditions or in the soil. This was also reflected in salt-stress treatments as they noted similar leaf proline distribution levels between the wild-type and *atprot3-2* and the authors concluded that the lack of the strong phenotype of the *atprot* triple knockout mutants lines is due to compensation by the other root-localized proline transporters (LHT1 and APP1). Our results also reflected this as significant deviations in PCD levels from wild-type seedlings only occurred when either LHT1 or AAP1 are inactivated. Phloem-localized AtProT1 is responsible for long-distance proline translocation and can be replaced by the AAP1 transporter, while AtProT2 is found in the root epidermis and imports extracellular proline into the root cortex; this can be replaced by both LHT1 and AAP1 transporters (Perchlik et al., 2014). Thus, our work provides reinforcing evidence of the functional overlap shared between ProT and other proline transporters (Lehmann et al., 2011).

Between exogenous proline and *N. muscorum* CM treatments, the latter resulted in the lowest stress-induced PCD levels across wild-type and mutant lines. The biological matrix likely contains additional bioactive compounds that act synergistically to exert a stronger PCD-suppressing effect than individual proline treatments alone. We did not confirm the identity of these additional compounds, but candidates include EPS, phytohormones, ROS-detoxifying exoproteins and phenolics. EPS such as arabinose, glucose, galactose, rhamnose, xylose and ribose have been detected in *N. muscorum* CM (Mehta and Vaidya, 1978) and carbohydrates are organic osmolytes that protect macromolecule structure against denaturing stress conditions (Yancey, 2005; Judy and Kishore, 2016). Microbial-derived phytohormones have been suggested to play an important role in plant survival fitness (Fahad et al., 2015; Egamberdieva et al., 2017). A substantial portion of *N. muscorum* exoproteins were associated with ROS detoxification, suggesting the importance of maintaining redox homeostasis even outside the cell (Oliveira et al., 2015). Moreover, the high concentrations of phenolics and alkaloids in *N. muscorum* CM may buffer against oxidative damage (El-Sheekh et al., 2006; Abdel-Hafez et al., 2015). Collectively, reduction of ROS damage may suppress activation of PCD-inducing signals.

## Conclusion

In this study, we provide evidence that cyanobacteria-derived proline suppressed PCD in *Arabidopsis* root hairs. By using the RHA to characterize *N. muscorum* CM bioactivity, we found that a major bioactive compound was thermostable and directly affecting PCD levels but not necrosis. Proline was identified as a potential candidate and strong evidence using the ninhydrin assay and HPLC suggest that proline is present in *N. muscorum* CM. Subsequent testing with exogenous proline showed a similar root hair stress-response profile with *N. muscorum* CM treatment (higher viability, lower PCD and unaffected necrosis levels), showing that both treatments altered the PCD sensitivity threshold. However, the lower PCD rates observed in *N. muscorum* CM treatment is likely because of synergistic interactions between additional thermolabile bioactive compounds. We provide additional evidence for proline as the bioactive compound using three proline transporter mutants (*lht1*, *aap1* and *atprot* triple knockout). Both general amino acid transporter mutants (*lht1* and *aap1*) displayed similar stress phenotypes to each other, with consistently higher PCD levels than wild-type seedlings at medium to high proline doses. All three mutant lines had higher PCD levels when treated with *N. muscorum* CM compared to wild-type seedlings, providing additional evidence for proline as an important bioactive compound present in *N. muscorum* CM. Data from the mutant lines also reinforce earlier findings that the accompanying bioactive compounds in *N. muscorum* CM were strongly inhibiting PCD over proline treatment alone, which warrants further research in the future. Collectively, this offers preliminary evidence of an unconventional biofertiliser method for inhibiting environmentally induced PCD, distinct from the known mechanisms in literature.

## Data Availability Statement

All datasets generated for this study are included in the manuscript/Supplementary Material.

## Author Contributions

AC designed and performed the majority of the experiments. OS prepared and captured the Supplementary F1 image. CN, PM, and CD conceived several of the preliminary hypotheses. LF and CD contributed to the discussion of the results. All authors reviewed and approved the final manuscript.

## Conflict of Interest

The authors declare that the research was conducted in the absence of any commercial or financial relationships that could be construed as a potential conflict of interest.
