# Supplementary material for: Cyanobacteria-Derived Proline Increases Stress Tolerance in Arabidopsis thaliana Root Hairs by Suppressing Programmed Cell Death
Source: Front Plant Sci. 2020 Dec 14;11:490075. doi: 10.3389/fpls.2020.490075 (PMC7768022; doi:10.3389/fpls.2020.490075)
Supplement: Supplementary file 1 [file Data_Sheet_1.docx]

Supplementary Material - Cyanobacteria-derived proline increases plant stress tolerance in *Arabidopsis* *thaliana* root hairs by suppressing programmed cell death

Supplementary Figure S1 – *A. thaliana* Col-0 trichoblast cells stained with Evans Blue viability stain showing the whole trichoblast cell is dead, and the cytoplasmic extension that forms the hair. Image collected at 400x magnification using Leica MC170 HD. White arrows indicate retraction of the protoplast in the root hair and also the trichoblast. Scale bar: 5 µm.


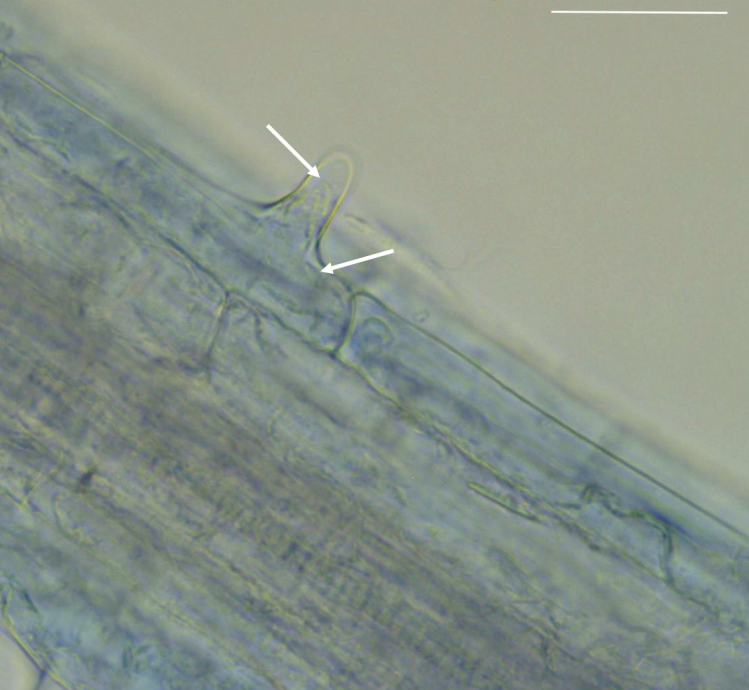


Supplementary Table S1 - Effect of autoclaved and non-autoclaved *N. muscorum* CM fractions on *Arabidopsis* root hair viability at 50°C heat stress. Values are the average of n ≥ 8 (± SE) and represent the merged results of 3 experiments. A Dunnett t-test was used for statistical analysis which treated the BG11 dataset as a control and compared all other group datasets against it. (*) The mean difference is significant at the 0.05 level.

| ***N. muscorum* CM Treatments** | | **PCD (%)** | **Mean difference from BG11 control (%)** | ***p*-value** |
| --- | --- | --- | --- | --- |
| Controls | BG11 | 75.1 ± 2.93 | N/A | N/A |
|  | SDW | 78.7 ± 2.51 | 3.6 | 0.989 |
| 20% | Non-autoclaved | 60.3 ± 2.66 | -18.5* | 0.001 |
|  | Autoclaved | 67.0 ± 3.73 | -11.8 | 0.079 |
| 40% | Non-autoclaved | 50.5 ± 3.09 | -28.3^*^ | 0.000 |
|  | Autoclaved | 61.2 ± 3.51 | -17.5* | 0.002 |
| 60% | Non-autoclaved | 42.4 ± 2.48 | -36.3^*^ | 0.000 |
|  | Autoclaved | 54.9 ± 3.31 | -23.9^*^ | 0.000 |
| 80% | Non-autoclaved | 38.1 ± 3.01 | -40.7^*^ | 0.000 |
|  | Autoclaved | 46.3 ± 3.01 | -32.4^*^ | 0.000 |
| 100% | Non-autoclaved | 41.2 ± 2.54 | -37.5^*^ | 0.000 |
|  | Autoclaved | 56.0 ± 4.64 | -22.7^*^ | 0.000 |

Supplementary Table S2 - Effect of autoclaved and non-autoclaved exogenous proline on *Arabidopsis* root hair viability at 50°C heat stress. Values are the average of n ≥ 12 (± SE) and represent the merged results of 3 experiments. A Dunnett t-test was used for statistical analysis which treated the BG11 dataset as a control and compared all other group datasets against it. (*) The mean difference is significant at the 0.05 level.

| **Proline Treatments** | | **PCD (%)** | **Mean difference from BG11 control (%)** | ***p*-value** |
| --- | --- | --- | --- | --- |
| Controls | BG11 | 66.5 ± 2.61 | N/A | N/A |
| 20% | Non-autoclaved | 48.9 ± 2.43 | -17.6* | 0.002 |
|  | Autoclaved | 44.3 ± 2.12 | -22.2* | 0.000 |
| 40% | Non-autoclaved | 42.6 ± 4.62 | -24.0* | 0.000 |
|  | Autoclaved | 47.7 ± 2.75 | -18.9* | 0.000 |
| 60% | Non-autoclaved | 51.3 ± 3.35 | -15.2* | 0.006 |
|  | Autoclaved | 43.6 ± 3.12 | -22.9* | 0.000 |
| 80% | Non-autoclaved | 42.9 ± 2.75 | -23.6* | 0.000 |
|  | Autoclaved | 50.4 ± 3.51 | -16.1* | 0.005 |
| 100% | Non-autoclaved | 44.9 ± 5.36 | -21.6* | 0.000 |
|  | Autoclaved | 51.0 ± 2.63 | -15.6* | 0.027 |

Supplementary Table S3 – Comparing stress-induced PCD levels between autoclaved (AC) and non-autoclaved *N. muscorum* CM and exogenous proline in heat shocked *Arabidopsis* seedlings. Samples were diluted in BG11 across a concentration gradient (0-100%). Solutions labelled ‘100% proline’ corresponded to proline levels previously measured in undiluted *N. muscorum* CM. The remaining gradient (20-80%) was established in BG11, where 80% proline = 80% proline + 20% BG11, 60% proline = 60% proline + 40% BG11, etc. Values for each dataset represent the average of n ≥ 8 (± SE) and represent the merged results of 3 experiments. A one-way ANOVA Tukey post-hoc test was used for statistical analysis. (*) The mean difference is significant at the 0.05 level.

| **20% Treatment** | **Non-Autoclaved CM** | | | | | **Autoclaved CM** | | | | | **Autoclaved proline** | | |
| --- | --- | --- | --- | --- | --- | --- | --- | --- | --- | --- | --- | --- | --- |
|  | Autoclaved CM | | Non-autoclaved proline | | Autoclaved proline | Non-autoclaved proline | | | Autoclaved proline | | Non-autoclaved proline | | |
| Mean Difference | -6.69 | | 11.4* | | 16.0* | 18.1* | | | 22.7* | | -4.61 | | |
| Std. Error | 3.87 | | 4.04 | | 3.95 | 4.17 | | | 4.08 | | 4.24 | | |
| Sig. | 0.321 | | 0.034 | | 0.001 | 0 | | | 0 | | 0.699 | | |
|  |  |  | |  | | |  |  | |  | |  |  |
| **40% Treatment** | **Non-Autoclaved CM** | | | | | **Autoclaved CM** | | | | | **Autoclaved proline** | | |
|  | Autoclaved CM | | Non-autoclaved proline | | Autoclaved proline | Non-autoclaved proline | | | Autoclaved proline | | Non-autoclaved proline | | |
| Mean Difference | -10.78 | | 7.89 | | 2.8 | 18.7* | | | 13.6* | | 5.09 | | |
| Std. Error | 4.94 | | 5.04 | | 4.85 | 5.04 | | | 4.85 | | 4.95 | | |
| Sig. | 0.143 | | 0.407 | | 0.938 | 0.003 | | | 0.036 | | 0.734 | | |

| **60% Treatment** | **Non-Autoclaved CM** | | | | | | **Autoclaved CM** | | | | | **Autoclaved proline** | | |
| --- | --- | --- | --- | --- | --- | --- | --- | --- | --- | --- | --- | --- | --- | --- |
|  | Autoclaved CM | | | Non-autoclaved proline | | Autoclaved proline | Non-autoclaved proline | | | Autoclaved proline | | Non-autoclaved proline | | |
| Mean Difference | -12.4* | | | -8.9 | | -1.23 | 3.54 | | | 11.21 | | -7.67 | | |
| Std. Error | 4.33 | | | 4.33 | | 4.58 | 4.25 | | | 4.51 | | 4.51 | | |
| Sig. | 0.029 | | | 0.181 | | 0.993 | 0.839 | | | 0.074 | | 0.334 | | |
|  | |  | | | |  |  |  |  |  |  |  |  |  |
| **80% Treatment** | **Non-Autoclaved CM** | | | | | | **Autoclaved CM** | | | | | **Autoclaved proline** | | |
|  | Autoclaved CM | | | Non-autoclaved proline | | Autoclaved proline | Non-autoclaved proline | | | Autoclaved proline | | Non-autoclaved proline | | |
| Mean Difference | -8.23 | | | -4.84 | | -12.34 | 3.4 | | | -4.11 | | 7.5 | | |
| Std. Error | 4.81 | | | 4.6 | | 4.73 | 4.23 | | | 4.37 | | 4.14 | | |
| Sig. | 0.33 | | | 0.72 | | 0.058 | 0.853 | | | 0.783 | | 0.28 | | |
|  |  | |  | |  | | |  |  | |  | |  |  |
| **100% Treatment** | **Non-Autoclaved CM** | | | | | | **Autoclaved CM** | | | | | **Autoclaved proline** | | |
|  | Autoclaved CM | | | Non-autoclaved proline | | Autoclaved proline | Non-autoclaved proline | | | Autoclaved proline | | Non-autoclaved proline | | |
| Mean Difference | -14.79 | | | -3.7 | | -9.76 | 11.09 | | | 5.03 | | 6.06 | | |
| Std. Error | 5.57 | | | 5.81 | | 6.35 | 5.7 | | | 6.26 | | 6.47 | | |
| Sig. | 0.053 | | | 0.92 | | 0.426 | 0.226 | | | 0.852 | | 0.785 | | |

Supplementary Table S4 - Effect of exogenous proline and *N. muscorum* CM on *Arabidopsis* root hair viability at 50°C heat stress in wild-type and amino acid transporter mutants (*atprot1-1*::*atprot2-3*::*atprot3-2*, *aap1* and *lht1*). Values are the average of n ≥ 12 (± SE) and represent the merged results of 3 experiments. (*) marks PCD levels significantly (*p*<0.05) different from their respective BG11 datasets as a control, using a one-way ANOVA Dunnett post-hoc test.

| **Treatment** | | **PCD (%)** | **Mean difference from BG11 control (%)** | ***p*-value** |
| --- | --- | --- | --- | --- |
| Wild-type | BG11 | 48.1 ± 2.92 | N/A | N/A |
|  | SDW | 52.7 ± 2.30 | 4.57 | 0.572 |
|  | 1 μM proline | 37.6 ± 2.77 | -10.5^*^ | 0.041 |
|  | 2 μM proline | 44.2 ± 3.08 | -3.94 | 0.862 |
|  | 5 μM proline | 35.7 ± 2.54 | -12.4^*^ | 0.010 |
|  | 100 μM proline | 44.5 ± 3.15 | -3.58 | 0.894 |
|  | 100% CM proline | 26.7 ± 1.67 | -21.4^*^ | 0.000 |
| *atprot1-1::atprot2-3::atprot3-2* | BG11 | 47.3 ± 3.73 | N/A | N/A |
|  | SDW | 46.4 ± 4.63 | -0.97 | 1.000 |
|  | 1 μM proline | 43.5 ± 3.47 | -3.82 | 0.969 |
|  | 2 μM proline | 43.4 ± 5.22 | -3.97 | 0.963 |
|  | 5 μM proline | 45.2 ± 4.64 | -2.09 | 0.999 |
|  | 100 μM proline | 48.7 ± 3.78 | 1.33 | 1.000 |
|  | 100% CM proline | 35.4 ± 3.00 | -11.9 | 0.170 |
| *aap1* | BG11 | 48.4 ± 2.54 | N/A | N/A |
|  | SDW | 55.1 ± 5.81 | 6.72 | 0.623 |
|  | 1 μM proline | 41.6 ± 2.43 | -6.85 | 0.585 |
|  | 2 μM proline | 57.4 ± 2.98 | 9.02 | 0.329 |
|  | 5 μM proline | 55.4 ± 3.35 | 6.96 | 0.570 |
|  | 100 μM proline | 59.5 ± 3.59 | 11.12 | 0.153 |
|  | 100% CM proline | 39.0 ± 4.33 | -9.37 | 0.293 |
| *lht1* | BG11 | 53.1 ± 2.82 | N/A | N/A |
|  | SDW | 55.5 ± 3.30 | 2.37 | 0.994 |
|  | 1 μM proline | 50.0 ± 4.32 | -3.11 | 0.975 |
|  | 2 μM proline | 51.0 ± 2.92 | -2.11 | 0.997 |
|  | 5 μM proline | 55.0 ± 3.41 | 1.83 | 0.999 |
|  | 100 μM proline | 58.0 ± 4.22 | 4.83 | 0.849 |
|  | 100% CM proline | 37.6 ± 3.71 | -15.6^*^ | 0.014 |

Supplementary Table S5 - Effect of exogenous proline and *N. muscorum* CM on *Arabidopsis* root hair viability at 50°C heat stress in wild-type and amino acid transporter mutants (*atprot1-1*::*atprot2-3*::*atprot3-2*, *aap1* and *lht1*). (*) marks PCD levels significantly (*p*<0.05) different from the WT datasets as a control, using a one-way ANOVA Dunnett post-hoc test. Values are the average of n ≥ 12 (± SE) and represent the merged results of 3 experiments. *N. muscorum* CM properties: OD730 (1.43), chl-a (18.9 µg/ml) and carotenoid (4.67 µg/ml)

| **Treatment** | **Genetic lines** | **PCD (%)** | **Mean difference from WT control (%)** | ***p*-value** |
| --- | --- | --- | --- | --- |
| BG11 | WT | 48.1 ± 2.92 | N/A | N/A |
|  | *atprot1-1::atprot2-3::atprot3-2* | 47.3 ± 3.73 | -0.76 | 0.996 |
|  | *aap1* | 48.4 ± 2.54 | 0.32 | 1.000 |
|  | *lht1* | 53.1 ± 2.82 | 5.06 | 0.549 |
| 1 μM pro | WT | 37.6 ± 2.77 | N/A | N/A |
|  | *atprot1-1::atprot2-3::atprot3-2* | 43.5 ± 3.47 | 5.94 | 0.461 |
|  | *aap1* | 41.6 ± 2.43 | 3.98 | 0.728 |
|  | *lht1* | 50.0 ± 4.32 | 12.5^*^ | 0.027 |
| 2 μM pro | WT | 44.2 ± 3.08 | N/A | N/A |
|  | *atprot1-1::atprot2-3::atprot3-2* | 43.4 ± 5.22 | -0.79 | 0.998 |
|  | *aap1* | 57.4 ± 2.98 | 13.3^*^ | 0.038 |
|  | *lht1* | 51.0 ± 2.92 | 6.88 | 0.416 |
| 5 μM pro | WT | 35.7 ± 2.54 | N/A | N/A |
|  | *atprot1-1::atprot2-3::atprot3-2* | 45.2 ± 4.64 | 9.52 | 0.153 |
|  | *aap1* | 55.4 ± 3.35 | 19.7^*^ | 0.001 |
|  | *lht1* | 55.0 ± 3.41 | 19.3^*^ | 0.001 |
| 100 μM pro | WT | 44.5 ± 3.15 | N/A | N/A |
|  | *atprot1-1::atprot2-3::atprot3-2* | 48.7 ± 3.78 | 4.15 | 0.764 |
|  | *aap1* | 59.5 ± 3.59 | 15.0^*^ | 0.015 |
|  | *lht1* | 58.0 ± 4.22 | 13.5^*^ | 0.033 |
| *N. muscorum* CM | WT | 26.7 ± 1.67 | N/A | N/A |
|  | *atprot1-1::atprot2-3::atprot3-2* | 35.4 ± 3.00 | 8.66 | 0.175 |
|  | *aap1* | 39.0 ± 4.33 | 12.3^*^ | 0.032 |
|  | *lht1* | 37.6 ± 3.71 | 10.8 | 0.068 |
